# Innovation in primary health care responses to COVID-19 in Sub-Saharan Africa

**DOI:** 10.1017/S1463423621000451

**Published:** 2021-09-15

**Authors:** Sunanda Ray, Robert Mash

**Affiliations:** 1 Extraordinary Professor Department of Medical Education, University of Botswana, Gaborone, Botswana; 2 Visiting Professor School of Public Health, University of Witwatersrand, Johannesburg, South Africa; 3 Distinguished Professor and Executive Head Division of Family Medicine and Primary Care, Faculty of Medicine and Health Sciences, Stellenbosch University, Stellenbosch, South Africa

**Keywords:** Africa region, community-oriented primary care, COVID-19, public health

## Abstract

**Background:**

In May 2020, the African Journal of Primary Health Care and Family Medicine invited submissions on lessons learnt from responses to the COVID-19 pandemic from primary care providers in Africa. This included descriptions of innovations and good practices, the management of COVID-19 in district health services and responses of communities to the outbreak.

**Aim:**

To synthesise the lessons learnt from the COVID-19 pandemic in the Africa region.

**Methods:**

A thematic document analysis was conducted on twenty-seven short report publications from Botswana, Ghana, Nigeria, South Africa, Uganda and Zimbabwe. *Findings:* Eight major themes were derived from the data: community-based activities; screening and testing; reorganisation of health services; emergency care for COVID-19; maintenance of essential non-COVID-19 health services; caring for the vulnerable; use of information technology; and reframing training opportunities. Community health workers were a vital community resource, delivering medications and other supplies to homes, as well as following up on patients with chronic conditions. More investment in community partnerships and social mobilisation was proposed. Difficulties with procurement of test kits and turn-around times were constraints for most countries. Authors described how services were reorganised for focused COVID-19 activities, sometimes to the detriment of essential services and training of junior doctors. Innovations in use of internet technology for communication and remote consultations were explored. The contribution of family medicine principles in upholding the humanity of patients and their families, clear leadership and planning, multidisciplinary teamwork and continuity of care was emphasised even in the context of providing critical care.

**Conclusions:**

The community-orientated primary care approach was emphasised as well as long-term benefits of technological innovations. The pandemic exposed the need to deliver on governmental commitments to strengthening primary health care and universal health coverage.

## Introduction

Coronavirus disease 2019 (COVID-19) is a major challenge to African health systems due to the scarcity of healthcare workers, lack of intensive and high care facilities, few public health specialists as well as weak primary health care (PHC) systems. The pandemic has not been as severe as feared in terms of mortality and hospital admissions, although confirmed cases may be low due to a lack of testing capacity, disguising the true extent of the pandemic (Bamgboye *et al.*, 2020). A household survey of six districts in Zambia showed a 100-fold higher level of COVID-19 infections than cases reported through the official national statistics, indicating a high level of hidden infections (Mulenga *et al.*, 2021). Some countries, such as South Africa, have reported rates on par with Europe and the USA. African populations may have fewer of the risk groups seen in high-income countries, such as the elderly and those with non-communicable diseases, but have other potential risk factors, such as HIV and TB (Dyer, 2020). In addition, widespread poverty and overcrowding in informal settlements and urban slums make isolation, quarantine and lockdown very difficult (Corburn *et al.*, 2020). Many African countries were used to responding to epidemics, such as Ebola, and may have been more experienced in handling outbreaks of infectious diseases than other countries (Bamgboye *et al.*, 2020).

The Astana Declaration, with its goal of universal health coverage (UHC), endorses a vision of sustainable PHC embedded in strong health systems, with three components. These are first, primary care and public health functions as the core of integrated health services, including health promotion, surveillance, preparedness and response for health emergencies and outbreaks; second, empowered people and communities, for whom health literacy and active participation in decisions about their health are facilitated; and third, multisectoral policy and coordinated action across sectors to achieve health goals and reduce health threats (Rasanathan & Evans, 2020).

For COVID-19 responses to be aligned with the Astana Declaration, interdisciplinary collaboration between primary care, public health, community-based structures, emergency medicine and referral mechanisms is essential. Effective emergency preparedness requires urgent and responsive communication and coordination between centralised planners, policymakers and managers, with their implementation partners at primary care levels (Garg *et al.*, 2020). A review of guidelines during January to April 2020 from 14 countries identified public health guidance on outbreak control measures, use of telehealth and telephone triage to ensure continued delivery of essential primary care services and integrated planning for training, capacity building and data sharing with partners from various sectors (Haldane *et al.*, 2020). Guidance from some countries recommended involvement of community networks and organisations, especially for high-risk groups, including use of community health workers (CHWs) to create supportive local environments and facilitate contact tracing and case finding. Community structures were also identified as resources for delivering food, medication and other goods, especially for home-bound patients and their families. Gaps in guidance included mechanisms for ensuring adequate provision of personal protective equipment (PPE), testing kits and supply chain management. The authors suggest that strong partnerships between primary care and public health are essential for effective testing, contact tracing and support services. Communication and coordination between primary and secondary care services also need strengthening (Haldane *et al.*, 2020).

Other authors commented that many countries were unprepared at the onset of the COVID-19 pandemic in service reorganisation, COVID-19 screening and testing, and ensuring other essential services continued. Community cadres were bypassed in surveillance and health promotion activities which were often instituted centrally, and restrictions limiting travel and access to primary care facilities undermined the role of primary care as the first line in healthcare for communities (Rasanathan & Evans, 2020). Reviews of how primary care systems coped at the onset of the COVID-19 pandemic revealed not only critical weaknesses in health systems and intersectoral responses but also rapid adaptations in response to new challenges (Allen & Dambha-Miller, 2020). In well-resourced countries with already functioning UHC, the initial assessment and triage of people suspected of having COVID-19 was carried out by primary care teams, sometimes in assessment centres or remotely by telephone, email and videoconferencing (Huston *et al.*, 2020). In many countries, primary care facilities were reorganised to prioritise COVID-19 patients and to reduce risk of transmission, especially to vulnerable people (Chang & Chiu, 2020; Garg *et al.*, 2020; Huston *et al.*, 2020; Lim & Wong, 2020; Sarti *et al.*, 2020).

Information dissemination through daily press briefings, civic education and clear guidelines boosted confidence in government agencies. Such actions were proactively implemented at early stages of the pandemic by Taiwan and Singapore, who built on lessons learnt from the 2003 SARS (severe acute respiratory syndrome) and 2009 Influenza H1N1 epidemics (Chang & Chiu, 2020; Lim & Wong, 2020). In countries like Brazil and India, these adaptations were sometimes limited by inflexible infrastructure and poor coordination (Garg *et al.*, 2020; Sarti *et al.*, 2020). Brazil, albeit with an extensive primary care network and established UHC, struggled with difficulties in funding, personnel, supply and service restructuring (Sarti *et al.*, 2020).

Telemedicine, video and zoom consultations rapidly expanded and became more accessible to the public (Huston *et al.*, 2020; Sarti *et al.*, 2020). Reviews stressed the importance of already-established public health surveillance systems to ensure real-time data were collected to monitor emerging symptoms, complications, access to services, testing results and actions (Huston *et al.*, 2020). External and internal travel restrictions, border controls, as well as testing and home isolation or quarantine arrangements, required prior pandemic planning and legislative frameworks for rapid implementation.

Public understanding of the urgent need for restrictive public health measures such as physical distancing, mask-wearing, travel restrictions, hand hygiene and homeworking was essential. Provision of PPE and testing/contact-tracing services were slow to be established and suffered from competition in global markets for supplies (Garg *et al.*, 2020). Decreased use of primary care services for non-communicable diseases (NCDs), disruptions of other essential primary care services such as maternal and child care (MCH), HIV and tuberculosis services, with reduced referral for investigations and specialist assessments, led to fears of increased morbidity and mortality from non-COVID-19 illnesses (Garg *et al.*, 2020).

As the pandemic gained traction in Africa during May 2020, the African Journal of Primary Health Care and Family Medicine (PHCFM)^1^ called for primary care providers and family physicians from countries in Africa to submit short reports on how they were responding and what they were learning about COVID-19. We wanted to look for opportunities to prepare for the pandemic and to make the most of the period between the early cases and widespread community transmission, for training health staff, reorganising health facilities, exploring strategic approaches that describe clear roles, tasks and communications, and to develop ways of communities supporting each other (Ray, 2020). We asked for descriptions of innovations and good practices in PHC and family medicine (FM), including the contributions of family physicians, the management of COVID-19 in district health services and responses of communities to the outbreak.

These short reports were descriptive and do not present empirical data. They did not report on research or provide definitive evidence of effectiveness of the ideas presented. They did not attempt to be representative of the total experience of COVID-19 services in Africa and were limited to authors who chose to respond to the journal’s call for short reports. Each short report was peer-reviewed by one or two reviewers. The aim of this paper is to synthesise the lessons described in these short reports and to guide readers to ideas and resources available in addressing the challenges raised by the COVID-19 pandemic.

## Methods

This was a qualitative descriptive study of short reports published in PHCFM with a thematic analysis. All short reports accepted for publication in a special collection on COVID-19 were included in the qualitative analysis. The analysis was conducted by the two authors, SR and RM. SR is an assistant editor of PHCFM and oversaw the special edition; RM is the Editor-in-Chief of PHCFM and also an author on some of the short reports. The reports were initially divided between the two authors, who independently analysed their allocated documents. RM used Atlas-ti software, while SR carried out the analysis manually. Each conducted the analysis using the following steps: reading the reports to familiarise with the contents, coding all the documents inductively, creating coding groups and charts based on these groups that brought all the same coded data together and finally interpreting the data in the charts. The two researchers then discussed their analyses and came to a consensus on the code groups and themes they had derived. They reread all twenty-seven papers and integrated their analyses into one set of themes and analysis which are presented here.

Ethics approval and participant consent were not required since all the papers reviewed were published in the journal and in the public domain.

## Findings

Twenty-seven short reports were accepted for publication in the PHCFM and were submitted from six countries: Botswana, Ghana, Nigeria, South Africa, Uganda and Zimbabwe (see Table 1). The majority of short reports published were from South Africa, reflecting the proportion of submissions to the journal in general. South Africa is a leading country in terms of academic outputs and family medicine in the region. There were eight main themes derived from the data: community-based activities; screening and testing; reorganisation of health services; provision of emergency care for COVID-19; maintenance of essential non-COVID-19 health services; caring for the vulnerable; use of information technology and reframing training opportunities.

**Table 1.** Summary of submissions published in special issue of Primary Health Care and Family Medicine on COVID-19 May–August 2020

Title & First Author	Discipline	Country	Type of report
FD Madzimbamuto *Ventilators are not the answer in Africa*	Anaesthesia and Critical Care	Botswana	Opinion paper on need for access to oximeters, oxygen concentrators and oxygen production rather than ventilators for most hospitals in countries in Africa; highlighted_resources available for district hospitals to manage moderate to severe COVID-19 illness
K Motlhatlhedi *et al.* *Coronavirus disease 2019 in Botswana: Contributions from family physicians.*	Family Medicine	Botswana	Practical examples of ways primary care services have been restructured to allow for screening of people with possible COVID-19 infection are given.
B Tsima *et al.* *Service-learning in response to the coronavirus disease 2019 pandemic:*	Family Medicine	Botswana	Description of how training in family medicine and public health medicine has been re-structured to accommodate learning opportunities related to the COVID-19 pandemic
NK Ayisi-Boateng *et al.* *Exploring the illness experiences amongst families living with 2019 coronavirus disease in Ghana: Three case reports.*	Family Medicine	Ghana	The biopsychosocial impact of COVID-19, the illness experiences and fears of three families are described, of death, stigmatisation, family separation and loss of income. The role of family physicians and primary care in addressing these challenges is discussed.
P Vandyck-Sey *et al.* *Incidental finding of COVID-19 infection amongst staff at a primary care facility in Ghana.*	Family Medicine	Ghana	The authors described how screening health staff for COVID-19 and the discovery of several asymptomatic positive cases led to changes in clinical behaviour and use of PPE
O Olagundoye *et al.* *Recommendations for a national Coronavirus disease 2019 response guide-line for the care of older persons in Nigeria during and post-pandemic: A family physician’s perspective.*	Family Medicine	Nigeria	The authors recommend actionable strategies to prevent COVID-19-related morbidity or mortality among older persons in Nigeria and to promote their overall well-being during and after the pandemic. The recommendations cut across the domains of physical health, mental health, functioning ability and socio-environmental situation.
TIA Oseni et al *The role of the family physician in the fight against Coronavirus disease 2019 in Nigeria*.	Family Medicine	Nigeria	Measures include triaging patients at clinics; health education; social distancing, regular handwashing and compulsory use of face mask by both physicians and patients during clinical consultations, family-focused behavioural interventions, home-based care, telemedicine and palliative care services to the elderly and terminally ill.
AA Adeniji *Self-collected upper respiratory tract swabs for COVID-19 test*	Family Medicine	South Africa	Self-testing with nasopharyngeal swabs is proposed as a way of increasing the testing rate, while preserving scarce PPE and protecting health staff from exposure to infection. The feasibility of this was not evaluated.
CL Barrett *Primary healthcare practitioners and patient blood management in Africa in the time of coronavirus disease 2019*	Clinical Medicine	South Africa	The three pillars of patient blood management (optimise erythropoiesis, minimise blood loss and bleeding, plan and optimise physiological reserve of anaemia) is described, with the role of primary healthcare practitioners in sustaining the resilience of the blood supply during a crisis such as the COVID-19 pandemic.
A Booth *et al.* *Analysis of a SARS-CoV-2 daily screening programme for healthcare workers at a district hospital in KwaZulu-Natal, a quality improvement initiative.*	Family Medicine	South Africa	The South African Department of Health requires that all employees be screened daily for symptoms and potential persons under investigation identified timeously. This report assessed the usefulness of daily self-screening tools in detecting and managing potential health worker cases of COVID-19, in a hospital situated in KwaZulu-Natal, consisting of questions on symptoms and epidemiological risk factors.
Z Brey *et al.* *Home delivery of medication during Coronavirus disease 2019, Cape Town, South Africa: Short report*.	Family Medicine	South Africa	This paper describes the process of home delivery of medications set up by the Metropolitan Health Services in Cape Town. The chronic diseases dispensing system was linked with community-orientated primary care, through primary care pharmacies, local non-profit organisations and a city-wide network of community health workers.
N David *et al.* *Community-based screening and testing for Coronavirus in Cape Town, South Africa: Short report.*	Family Medicine	South Africa	The authors describe the practical steps taken to set up community-based screening and testing in Cape Town, using CHWs to administer household symptomatic screening questionnaires, to identify people who needed PCR testing. The difficulties faced in implementation to achieve the goals of the programme are explained.
PP Furstenburg *et al.* *Emergency centre reorganization in preparation to the COVID-19 pandemic: A district hospital’s dynamic adaptation response*.	Emergency medicine & critical care	South Africa	This paper gives practical ideas how to reorganise staffing, patient-streaming and floor-space organisation of the emergency centre of a district hospital to accommodate the surge of patients, improve infection control and prevent nosocomial transmission of COVID-19.
LS Jenkins *et al.* *The evolving role of family physicians during the coronavirus disease 2019 crisis: An appreciative reflection*.	Family & emergency medicine	South Africa	The authors in this paper discuss how to turn disruption of services and training into opportunities to be innovative during the COVID-19 crisis. They emphasise the importance of preventative and promotive care and using community-based approaches, with family physicians embracing their roles as health team leaders, capacity builders and clinical trainers.
O Johnson *et al.* *Why communities must be at the centre of the Coronavirus disease 2019 response: Lessons from Ebola and HIV in Africa*	Health services research	South Africa	The authors refer to lessons learnt from previous epidemics. They recommend that communities should be supported to develop their own response plans, community leaders should be recognised as vital assets, community representatives should have equal inclusion in strategic meetings and greater empathy should be built into decision-making processes.
R Mash *et al.* *Re-organising primary health care to respond to the Coronavirus epidemic in Cape Town, South Africa*.	Family Medicine	South Africa	In this paper, the authors give practical examples of how the Metropolitan Health Services have reorganised their community-orientated primary care services to maintain physical distancing at facilities to reduce risk to people using the services, and the need to prepare for an influx of people with mild to moderate COVID-19 which could be managed in primary care. The paper includes some items from a facility readiness checklist adapted from the National Institute for Communicable Diseases.
MS Moolla *et al.* *Implementing a video call visit system in a coronavirus disease 2019 unit.*	General Medicine	South Africa	The authors described implementation of a low-cost video call visit system at Tygerberg Hospital, Cape Town, presenting the successful elements and potential pitfalls of this project in the context of a pandemic, notably cross-infection and privacy.
J Nyasulu *et al.* *The effects of coronavirus disease 2019 pandemic on the South African health system: A call to maintain essential health services.*	Clinical Medicine	South Africa	The authors applied the World Health Organisation health systems framework and its six building blocks to assess how the COVID-19 pandemic had impacted the South African health system. They provide a framework of possible solutions to maintain essential health services while responding to the pandemic.
SO Onagbiye *et al.* *Novel coronavirus and regular physical activity involvement: Opinion*	Sport exercise recreation	South Africa	This article suggests breathing exercises and physical activities that could be beneficial in preventing unwanted weight gain as well as associated conditions such as anxiety and depression resulting from the lockdown in South Africa.
JD Porter *et al.* *Turnaround times – the Achilles’ heel of community screening and testing in Cape Town, South Africa: A short report*.	Family Medicine & Virology	South Africa	The difficulties faced in implementing community screening and testing (CST) in Cape Town are detailed with lessons for other countries trying to implement the same policy. The reasons for the prolonged turnaround times and delays in giving results are discussed, and the implications related to quarantine and work.
S Reid *et al.* *The Cape Town International Convention Centre from the inside: The family physicians’ view of the ‘Hospital of Hope’*.	Primary Health Care	South Africa	The week-by-week reflections of a group of family physicians who joined the clinical and operational management teams tasked with providing the inpatient service of an 862-bed COVID-19 field hospital in Cape Town. Useful experiential points on setting up similar field hospitals are given as well as particular attributes of family medicine in providing clinical care in this environment.
H Reuter *et al.* *Prohibiting alcohol sales during the coronavirus disease 2019 pandemic has positive effects on health services in South Africa*.	Primary Healthcare	South Africa	The impact of the alcohol ban during lockdown in SA is discussed in terms of reduced trauma, vehicle accidents and violence; that this reduced pressure on emergency services in Cape Town so that they could concentrate on dealing with COVID-19 patients. The possible benefits of longer term prohibition during the pandemic are presented.
NV Roman *et al.* *Spiritual care – ‘A deeper immunity’ – A response to Covid-19 pandemic.*	Child and family studies	South Africa	Insights into the necessity of providing spiritual care as a means of coping and well-being for families, patients and healthcare workers during the COVID-19 pandemic.
TJ Sehoole *COVID-19: Pandemic burden in Sub-Saharan Africa and the right to health –*	Human rights & law	South Africa	Advocacy for key investment decisions to be made for human and environmental well-being, not profit; creation of joint private–public approaches to develop a unified strong regulatory capacity and infrastructure over the entire healthcare sector which monitors competitiveness and ensures adherence to human rights responsibilities.
G Solomon *et al.* *Family medicine internship support during the COVID-19 pandemic in Cape Town*	Family Medicine	South Africa	Development of the Vanguard Supervision Tool for Medical Interns (VaSTMI-COV19) using a modified Delphi approach, matching supervisory methods with educational outcomes and skills to be gained.
IK Besigye *et al.* *Coronavirus disease-2019 epidemic response in Uganda: The need to strengthen and engage primary healthcare*.	Family Medicine	Uganda	The response to the COVID-19 pandemic in Uganda with primary care opportunities and pitfalls described. The authors comment on the underutilisation of CHWs and village health teams in giving health education and addressing fears and stigma in communities.
T Kusotera *et al.* *Coronavirus-19 and malaria: The great mimics*	Internal Medicine	Zimbabwe	The use of SARS-CoV-2 rapid diagnostic test kits for screening has raised serious concerns over their role in malaria areas. Coupled with a lack of personal protective equipment and knowledge on the possible interaction between malaria and COVID-19 in terms of presentation and shared symptoms, many frontline primary care workers are fearful about their risks of infection.

### Community-based activities

The Metro Health Services (MHS) in Cape Town, as part of the Government of the Western Cape, embraced the concept of Community Orientated Primary Care (COPC) since 2017 in policy and reorganised PHC accordingly (David & Mash, 2020; Mash, Goliath & Perez, 2020). The principles of COPC involve delineation of geographic areas, alignment of these areas with primary care facilities and networks of CHW teams. There were approximately 2500 CHWs employed by multiple non-profit organisations. The MHS had a number of adaptive community-based responses to the COVID-19 outbreak, which were described in several articles by different authors (Brey *et al.*, 2020; David & Mash, 2020; Mash, Goliath & Perez, 2020; Porter, Mash & Preiser, 2020). CHWs provided domiciliary visits for follow-up of patients with HIV, TB, mental disorders, postnatal care and palliative care needs. They also home-delivered medications, non-pharmaceutical equipment and supplies to people with chronic conditions (184 000 parcels delivered in first month), thus protecting them from potential exposure at primary care facilities and in public transport (Brey *et al.*, 2020; Mash, Goliath & Perez, 2020).

Beyond these examples from Cape Town, there was little evidence of community-orientated responses, engagement or local priority setting in the other short reports. Some described public health education campaigns that were carried out through community organisations and social media (Motlhatlhedi *et al.*, 2020; Oseni *et al.*, 2020). National level health education and mass sensitisation of the population was carried out in Uganda at all levels, while district and community leaders in Uganda were responsible for enforcing bans on gatherings and funerals and ensuring that soap and water were available in public places (Besigye, Mulowooza & Namatovu, 2020). Community support was provided as donations by local non-government organisations (NGOs), religious groups and businesses in Uganda (Besigye, Mulowooza & Namatovu, 2020). In South Africa, provision of food parcels or vouchers for food and help with transport and communications was reported (Brey *et al.*, 2020).

The COVID-19 response focused mainly on central hospitals and critical care provision, with prevention measures in Uganda enforced by security and military personnel, which may have deterred real community engagement. The authors felt that PHC-led activities could have utilised village health teams and CHWs more, as well as private health providers, to educate the public, address stigma and fear of infection (Besigye, Mulowooza & Namatovu, 2020). Using the World Health Organization’s (WHO) Health Systems Framework, authors suggested ways in which providing services closer to communities could reduce the impact of the pandemic. These included integrating health education campaigns with other primary care messages and delegation of health promotion on primary care to lower level health workers (Nyasulu & Pandya, 2020).

Social mobilisation brings together societal and individual influences to raise awareness of and demand for healthcare and to effect social change (WHO, no date). Authors wrote from experiences of Ebola in West Africa and of people living with HIV in Zimbabwe that social mobilisation in such epidemics needed to be at the top of the agenda, rather than the bottom, and that trust and confidence have to be built with communities to secure their cooperation (Johnson & Goronga, 2020). Public health strategies need to take account of the social, religious, cultural and political dynamics that influence the uptake of health interventions, instead of imposing interventions on communities. More investment in community partnerships should lead to better outcomes. Stigma and fear of infection within communities or resistance to unfeasible interventions may otherwise paralyse action against the virus (Besigye, Mulowooza & Namatovu, 2020; Johnson & Goronga, 2020).

Punitive quarantine measures may result in people avoiding diagnosis and hiding from health services, especially when their livelihoods and their ability to pay rent and feed their families are threatened. Community leaders and representatives of affected people should be recognised as the experts on their lived realities and included in discussions to provide health education (Johnson & Goronga, 2020). One author stressed the need to ensure that human rights and the right to health were protected, especially for the most vulnerable and not left to the private health marketplace in African countries (Sehoole, 2020). Nation states are legally bound to ensure that privatisation of services is not a threat in such a crisis, to the availability, accessibility and quality of health services. The author suggested that more equitable and essential services for COVID-19 could be provided through the creation of a wealth tax especially targeting those who were making profits from the crisis (Sehoole, 2020). Other mechanisms were proposed for protecting families against catastrophic out-of-pocket health expenditure, such as through public–private regulation, national health insurance or incentives for private health insurers to absorb costs of more equitable healthcare distribution (Sehoole, 2020).

An apparent benefit of the alcohol prohibition during the first level lockdown in South Africa was a reduction in trauma and emergency admissions not only due to assaults, road traffic accidents, and other injuries related to alcohol misuse but also due to fewer vehicles on the road (Barrett, 2020; Reuter *et al.*, 2020). Concerns for the resilience of blood supplies due to less availability of donors, thereby jeopardising emergency obstetric and intensive care, led to proposals for patient blood management systems to ameliorate the impact of shortages of donor blood, including the role of primary care practitioners in sustaining blood supplies (Barrett, 2020). Data reported from South Africa showed reductions in contact crimes (murder, rape and assault) and in numbers of unnatural deaths (Reuter *et al.*, 2020). There were also negative health consequences associated with lockdown. These included depression, anxiety and increases in domestic violence from being isolated with abusers, as well as social consequences of loss of income, food insecurity and unrest (Ayisi-Boateng *et al.*, 2020; Onagbiye *et al.*, 2020; Oseni *et al.*, 2020).

### Screening and testing

In Cape Town, highly vulnerable communities were identified as those with low socio-economic status and overcrowding in informal settlements. These communities also had high proportions of people suffering from HIV, tuberculosis, hypertension, diabetes and chronic lung disease, which predisposed them to more severe COVID-19 illness. All households in those communities were screened using a symptom-based questionnaire. Targeted screening was used to focus on households around known cases in other vulnerable communities. CHWs assisted with screening of communities using a symptom-based questionnaire (David & Mash, 2020; Porter, Mash & Preiser, 2020).

Testing for COVID-19 was a major problem for most African countries because of difficulties in procuring test kits and the turn-around times (TAT) at laboratories. In Uganda, testing was limited by the number of laboratories available and tests were initially only performed in one central laboratory, with no plans for community-based sample collection (Besigye, Mulowooza & Namatovu, 2020). The global shortage of rapid test polymerisation chain reaction (RT-PCR) kits, intended for diagnosis of acute infections, meant that some low- to medium-income countries resorted to using cheaper antibody (RDT-AB) test kits for screening, followed by PCR if positive, despite the low sensitivity of antibody tests (Kusotera & Nhengu, 2020). In Zimbabwe, in high malaria prevalence areas, patients with malaria (which presents with symptoms similar to COVID-19) were suspected of having COVID-19, leading to panic on the part of health staff and communities. This was made worse if they were tested using RDT-AB and had a false positive result, but with a negative confirmatory PCR test only returning from a central laboratory days later (Kusotera & Nhengu, 2020).

The community screening and testing programme in Cape Town used mobile testing centres that collected specimens from household members who had screened positive for COVID-19 symptoms by CHWs (Porter, Mash & Preiser, 2020). This was a strategy intended to slow the spread of COVID-19 by targeting hotspots in vulnerable communities. It was however made obsolete by the increasing TAT (Mash, Goliath & Perez, 2020). In primary care facilities, the TAT could take up to 31 days and rendered efforts at community screening useless as cases and contacts could not be followed up in time.

CHWs were then asked to screen households in their area but only refer for testing symptomatic people aged over 55 years or with co-morbidities. While the National Health Laboratory Service in South Africa aimed at a TAT of 48 h, this was seldom achieved. District hospitals had a median TAT of 3 days, which was longer than the median length of stay, so hospitalised patients were treated as persons under investigation (PUI) without a definitive diagnosis. In this way, the approach moved from preventing local transmission to case finding in high risk people (Porter, Mash & Preiser, 2020). One author proposed the idea of self-testing with nasopharyngeal swabs as a way of increasing the testing rate, while preserving scarce PPE and protecting health staff from exposure to infection, though the feasibility of this was not evaluated (Adeniji, 2020).

### Reorganisation of health services

Infrastructure at primary care facilities and hospitals had to be rapidly reorganised. In primary care, this was done to decongest services, to reduce the risk of infection and free up capacity to respond to the expected surge of patients with respiratory problems (Furstenburg *et al.*, 2020; Mash, Goliath & Perez, 2020; Motlhatlhedi *et al.*, 2020; Vandyck-Sey *et al.*, 2020). A PHC facility readiness checklist was adapted from the National Institute for Communicable Diseases and used in Cape Town to assess the readiness of the PHC system across key inputs and service delivery domains. The checklist of 43 items assessed facility management, screening and streaming of patient flows, use of infrastructure, supply chains, health intelligence and communication (Mash, Goliath & Perez, 2020). Health staff were relocated from other services to COVID-19 screening and treatment locations (Motlhatlhedi *et al.*, 2020).

Facilities attempted to comply with essential infection control methods: hand washing/sanitisation, physical distancing, use of face masks and other PPE as appropriate (Motlhatlhedi *et al.*, 2020; Oseni *et al.*, 2020; Vandyck-Sey *et al.*, 2020). Most facilities attempted to separate patients into PUI with respiratory symptoms in a ‘hot’ stream and those without any symptoms into a ‘cold’ stream. In Cape Town, patients with minor ailments were treated and discharged as soon as possible in both streams (Mash, Goliath & Perez, 2020). Several primary care facilities rapidly constructed COVID-19 testing and treatment centres from prefabricated structures, containers and tents outside the entrance. In southern Africa, the first wave of the pandemic peaked during the winter months, which meant that outside spaces could no longer be used due to rain and cold temperatures (Furstenburg *et al.*, 2020; Mash, Goliath & Perez, 2020).

Authors explored methods of protecting health workers who were at significant risk of contracting COVID-19, especially when PPE was in short supply globally (Mash, Goliath & Perez, 2020; Vandyck-Sey *et al.*, 2020). In some settings, staff fears led to overuse of PPE, while in other settings PPE had to be re-used or innovated. Staff were reportedly more likely to be infected in non-clinical areas and in the ‘cold’ stream as they felt less at risk and relaxed their precautions. Older staff members and those with co-morbidities were shielded from working in high exposure areas (Mash, Goliath & Perez, 2020; Vandyck-Sey *et al.*, 2020). In many situations, frontline health workers became burnt out and overwhelmed so more caring for carers was necessary.

The South African Department of Health required employees to be screened daily for COVID-19 before commencement of duty. Authors reported from a hospital in KwaZulu-Natal that a self-administered symptom-screening tool was ineffective because of poor completion as well as inadequate awareness and training (Booth, Omed & Naidoo, 2020). Several staff had positive symptoms but did not document what action was taken as a result. The team advised that internal quality control, improved oversight by managers and addition of temperature checks could improve adherence. They recommended that local champions (advocates and influencers within health teams) and management needed to endorse, drive and monitor implementation of the screening process and that staff had to be motivated to change their practices. Training in the need for safety measures, physical distancing, avoiding unnecessary meetings or social gatherings during breaks, and providing transport for staff to reduce use of public transport, were other measures suggested by authors (Booth, Omed & Naidoo, 2020).

### Provision of emergency care for COVID-19

Hospital emergency centres had to constantly adapt to the needs and volume of patients, and in South Africa, field hospitals were established to cope with large numbers of patients. The Hospital of Hope was established within a matter of weeks as an intermediate care facility at the Cape Town International Convention Centre, with over 800 beds, all with piped oxygen (Reid, Ras & von Pressentin, 2020). The availability of oxygen was a key feature that enabled care to be more than just assisted isolation. The medical management and staff were drawn mostly from family physicians, whose values included upholding the humanity of their patients despite the mass processing of people, clear leadership and planning, close multidisciplinary teamwork, frequent communication (with team members, patients and their families), continuity of care, commitment, mutual trust and willingness to adapt and learn. The importance of family-centred, multidisciplinary and team-based approaches in responding to the challenges of COVID-19 care was echoed in Ghana especially in supporting families that have been separated from their loved ones and the mental health challenges arising from this (Ayisi-Boateng *et al.*, 2020).

The challenges of managing patients with severe illness at district hospitals, because central hospitals were overwhelmed, were also explored (Madzimbamuto, 2020). Instead of being preoccupied with finding or manufacturing ventilators, district hospitals could plan to utilise accessible resources more equitably, for example by establishing early warning systems for hypoxia, acquiring oximeters, oxygen concentrators and training health staff in fundamentals of critical care. Sources were publicised of appropriate technology that were already available, such as support for securing oxygen supplies through Assist International, and the Lifebox Scheme for oximeters, were promoted through international partnerships such as the World Federation of Societies of Anaesthesiologists (Madzimbamuto, 2020). These resources would continue to be useful in supporting emergency care in district hospitals for emergency surgical, obstetric and neonatal care.

### Maintenance of essential non-COVID-19 health services

Authors (Barrett, 2020; Nyasulu & Pandya, 2020) reminded us of the WHO definition of resilience as the inbuilt capacity of the system to sustain provision of essential health and health-related services even when challenged by outbreaks, disasters or other shocks. Health systems did not demonstrate resilience in this context since there was significant disruption to essential services, especially for MCH, HIV, tuberculosis and non-communicable diseases (NCDs). Public health advice to stay at home, to avoid public facilities and transport may have led to decreased uptake of essential primary care services even when they were available (Mash, Goliath & Perez, 2020). In Zimbabwe, authors described how stigma and fear of infection associated with COVID-19 prevented access to testing and treatment for malaria (Kusotera & Nhengu, 2020). The authors reminded us of the WHO warning that malaria deaths could double if the focus on COVID-19 disrupted interventions for malaria (World Health Organization, 2020). Health workers were diverted from their usual workplaces to work in isolation centres and for local task forces, causing a breakdown in routine services. In Botswana, MCH services were restricted to immunisations and nutrition care, with reduced antenatal care only for high-risk pregnancies (Motlhatlhedi *et al.*, 2020).

Authors described actions taken to maintain essential primary care services and to prevent excess mortality from neglect of other conditions especially NCDs (diabetes, hypertension), HIV, tuberculosis and malaria. Activities, for instance in Botswana, included extension of review periods for stable NCD patients and medication refills at pharmacies without prior doctors’ consultation (Motlhatlhedi *et al.*, 2020). The WHO Health Systems Framework was used in one study to assess critical markers of resilience of the health system, for example, the ability to offer basic health care to pregnant women, immunisation for children and treatment for people with HIV (Nyasulu & Pandya, 2020). Their suggested solutions to maintaining services, despite deployment of health staff to COVID-19 areas, were to integrate services, to utilise medical, nursing and clinical associate students in non-COVID-19 essential services, convert part-time contracts to full time, bring back retired health staff into service, and to explore other task shifting measures, such as co-opting NGO partners and non-health staff from other government departments. They also recommended that ways of acknowledging and appreciating the health workforce be explored, to encourage cohesiveness in working together.

### Caring for the vulnerable

The need for explicit strategies to identify disadvantage and inequalities, and guidelines on providing targeted services for vulnerable groups, was a priority for authors from Uganda, South Africa and Nigeria (Besigye, Mulowooza & Namatovu, 2020; Nyasulu & Pandya, 2020; Olagundoye, Enema & Adebowale, 2020). Arrangements were required for provision of food, medication refills for chronic conditions, psychological support and communication (Ayisi-Boateng *et al.*, 2020; Motlhatlhedi *et al.*, 2020; Olagundoye, Enema & Adebowale, 2020). There was particular concern for the impact of social isolation due to physical distancing on older persons, those with mental health conditions and those on medication for HIV or other chronic illnesses (Olagundoye, Enema & Adebowale, 2020).

Reorganisation of services to reduce risk to vulnerable people included remote access through the use of telemedicine and toll-free phone lines, home delivery of medication, reduced waiting times and triage, prioritised testing, prioritisation of welfare packages and community-based interventions from civil society organisations (Ayisi-Boateng *et al.*, 2020; Brey *et al.*, 2020; Nyasulu & Pandya, 2020; Olagundoye, Enema & Adebowale, 2020; Oseni *et al.*, 2020). Shielding of elderly family members at home could be done through changes in sleeping arrangements, especially keeping school-going children separate (Johnson & Goronga, 2020). Financial arrangements included identifying responsible adults to have power of attorney if family members were incapacitated in hospital. Health insurance packages needed amendment to include home-based or palliative care (Olagundoye, Enema & Adebowale, 2020).

### Use of information technology and social media

Several authors mentioned that telephone consultations, telemedicine and web-based platforms were used to keep patients from attending health facilities. This was more feasible for private sector patients with more reliable internet and cell phone connectivity (Ayisi-Boateng *et al.*, 2020; Motlhatlhedi *et al.*, 2020; Nyasulu & Pandya, 2020; Oseni *et al.*, 2020). Telephonic consultations were introduced in Nigeria as alternatives to face-to-face consultations and if a visit was essential, a telephonic appointment systems ensured patients did not crowd together at the same time (Oseni *et al.*, 2020).

Spiritual care was often limited as chaplains and religious leaders were not allowed to enter facilities. In hospitals, visitors were prohibited and staff wore full PPE when administering to sick patients with COVID-19. Although health professionals supported the provision of spiritual care especially in end-of-life situations, they often felt ill-equipped to deliver it. Information and communications technology contributed significantly to making the response more humane at the same time as being more effective. Solutions proposed included use of social media and video links for virtual family visits though this required high-speed internet access (Reid, Ras & von Pressentin, 2020; Roman, Mthembu & Roman, 2020). In one hospital in Cape Town, “video call champions” were appointed in each ward whose role included raising awareness of the video call system and taking primary responsibility for making calls. Family members could visit from home or at the hospital in a separate room via web-based video calls (Moolla *et al.*, 2020).

Solutions to managing lockdown were proposed and included web-based games and videos (for those who could afford internet), which assisted people to take physical exercise, prevent weight gain and maintain good psychological well-being (Onagbiye *et al.*, 2020). Such initiatives could also benefit people with NCDs such as diabetes, hypertension and respiratory conditions. A WhatsApp robot was used in Cape Town to allow people to request home delivery of medication and to verify their addresses (Brey *et al.*, 2020). Information dissemination via social media and WhatsApp messaging was used for patient groups and practitioners in Botswana and was especially useful for communication to rural practitioners (Motlhatlhedi *et al.*, 2020). WhatsApp audio messaging was also suggested to provide information on self-care to people with NCDs.

### Reframing training opportunities

The reorientation of health services caused disruption to the learning environments and clinical training of registrars in family medicine (Jenkins *et al.*, 2020; Tsima, Masupe & Setlhare, 2020). Many services were de-escalated or stopped and registrars re-deployed to tackle the pandemic. This meant that many of their planned training activities could not go ahead. Related examinations and research activities were stopped or postponed. Learning opportunities were refocused on opportunities presented by the response to the pandemic, particularly as family physicians were at the forefront of reorganising primary care services. Examples given were of closer intersectoral working, better resource management and cooperation with the private sector, shifting towards more health promotive and preventive community-based approaches (Jenkins *et al.*, 2020). Adapting in response to a crisis was a key skill to be learnt, recognising that over-centralising control may lose the opportunity to build capacity at district levels and for active experiential learning (Jenkins *et al.*, 2020). At one health centre, a novel supervision tool was created to maximise the opportunities for learning (Solomon *et al.*, 2020). Educational activities in Botswana and South Africa were adjusted to use more virtual learning platforms and to develop alternative learning outcomes. Learning outcomes related to epidemic preparedness, emergency planning, surveillance and infection control, development of patient management protocols, knowledge synthesis from emerging scientific literature or work for the Ministries of Health on strategy and policy development. Flexibility with timetables also allowed registrars to use elective and self-directed learning periods to catch up on care for other conditions, even using web-based platforms to do this (Jenkins *et al.*, 2020; Solomon *et al.*, 2020; Tsima, Masupe & Setlhare, 2020).

## Discussion

The experiences, learning and recommendations shared in these papers from sub-Saharan Africa are not from original research and represent a diverse range of snapshots of different responses to the pandemic from different African countries. This synthesis can only reflect what authors chose to submit to us for publication and may not be representative of all activities undertaken. The paucity of community-based innovations does not mean none were undertaken in the region, but that they were not written about or submitted to PHCFM. Examples of community responses to COVID-19 have been published elsewhere (Loewenson *et al.*, 2021) and include activities mentioned here: community support for symptom surveillance, mobilising for blood donations, uptake of testing and contact tracing, distribution of food and other materials, involvement of local leadership and use of social media and telemedicine to disseminate information. Definitive evidence-based recommendations based on these papers cannot be made, but a number of themes have emerged, with ideas and lessons for better emergency preparedness and collaborative working. The papers in this special collection form a resource for providers for ideas on how to adapt and improve PHC services even in a time of crisis.

Many of the short reports described interventions similar to those in the international literature, especially in use of information technology, partnership between formal health structures and community-based cadres, and the role of primary care facilities in reorganising services to cope with the immediate crisis and to protect vulnerable people. According to these short reports, many African countries did not have PHC at the forefront of their responses to COVID-19 but the strength of preceding PHC systems may have predicted the ability to adapt and respond appropriately. For example, in Cape Town, implementation of policy on reorganising PHC was only possible because of strong functional governance structures that extended to the facility level and home delivery of medication was only possible because CHW teams were previously established in all communities. Community and village health workers in many countries were underutilised as mediators with the public, a shortcoming recognised by many authors. The challenges of stigma and fear of infection repeatedly experienced in disease outbreaks need much greater understanding since these phenomena posed barriers to screening, testing and care for COVID-19. Family physicians emphasised the importance of adaptive management that did not overly centralise control and which built peoples’ capacity to innovate, improve the response to emergencies in an incremental way and learn actively from experience at the frontline. Such decentralised leadership, however, also relied on family physicians or other clinical leaders within the PHC teams in distributed health services.

The COVID-19 pandemic exposed the need for countries to focus on strengthening PHC for more effective responses. In line with the Astana Declaration (Rasanathan & Evans, 2020), the importance of the COPC approach, which deliberately integrates public health and primary care approaches as well as facility and community-based services, is emphasised as the foundation from which to adapt and respond to emerging crises. Integration of public health and primary care approaches, rapid implementation of emergency preparedness plans and use of population based health data were found to be more important than having strong primary care systems in tackling COVID-19, albeit at an early stage of the pandemic (Goodyear-Smith *et al.*, 2020). The commitment of this COPC approach to community and stakeholder engagement may also be key in developing appropriate interventions, messages and reducing fear and stigma. Leadership from family physicians and primary care practitioners in establishing rapid communications, coordination and delegation from centralised policymakers and managers appeared to facilitate more sustainable and equitable pandemic responses.

The COVID-19 pandemic also highlighted the catalytic effect of access to appropriate technology and equipment. Key examples include cell phone and internet-based communication, laboratory capacity and the need to focus on oxygen supply rather than ventilators at this level of care. Access to cell phone technology and the internet transformed the way that health services related to patients and organised themselves. Technology was used to communicate with, protect vulnerable people and provide health care as well as coordinate teams of health care workers. Information and research findings were interrogated and disseminated more rapidly than in the past through webinars, podcasts and social media. Services could also more easily source equipment, PPE and other supplies. The crisis provided opportunities for innovation, which could have value for longer term development of health service delivery (Brey *et al.* 2020; Besigye, Mulowooza, & Namatovu 2020; Jenkins *et al.* 2020; Madzimbamuto 2020; Oseni *et al.* 2020).

The contribution of PHC will also be essential in the medium to long term as vaccination programmes are set up and in following up people with so-called “long” COVID-19. PHC will have to be at the forefront of the recovery stage of this pandemic in re-establishing services that may have been neglected, especially for HIV, tuberculosis, MCH and NCDs. The long-term impact of COVID-19 will be seen through its effect on UHC. The control of other priority diseases will have deteriorated, and the economic effects of lockdown on the social foundation of society will also impact on health. The measurement of impact will need to extend beyond hospitalisations and deaths. As we start to turn the corner on controlling the COVID-19 pandemic, we will need to continue the fight for UHC and PHC.

## Conclusion

Lessons learnt from African family medicine and PHC systems in responding to the COVID-19 pandemic can be summarised under eight major themes: community-based services, screening and testing, reorganisation of health services, provision of emergency care for COVID-19, maintenance of essential non-COVID-19 services, caring for the vulnerable, use of information technology and reframing training opportunities. The community-orientated primary care approach was emphasised as well as long-term benefits of technological innovations. The COVID-19 pandemic exposed the need to deliver on governmental commitments to strengthening PHC and UHC.

## Footnotes

1
https://phcfm.org/index.php/phcfm

## References

[ref1] Adeniji AA (2020) “Self-collected upper respiratory tract swabs for COVID-19 test”: a feasible way to increase overall testing rate and conserve resources in South Africa. African Journal of Primary Health Care and Family Medicine 12, 1–4. doi: 10.4102/PHCFM.V12I1.2445. PMC7300941 32501019

[ref2] Allen LN and Dambha-Miller H (2020) COVID-19 and international primary care systems: rebuilding a stronger primary care. BJGP Open 4. doi: 10.3399/bjgpopen20x101130. PMC7606142 32900706

[ref3] Ayisi-Boateng NK, Egblewogbe DA, Owusu-Antwi R, Essuman A and Spangenberg K (2020) Exploring the illness experiences amongst families living with 2019 coronavirus disease in Ghana: three case reports. African Journal of Primary Health Care & Family Medicine 12, a2682. doi: 10.4102/phcfm.v12i1.2682. PMC7670005 33181874

[ref4] Bamgboye EL, Omiye JA, Afolaranmi OJ, Davids MR, Tannor EK, Wadee S, Niang A, Were A and Naicker S (2020) COVID-19 pandemic: is Africa different? Journal of the National Medical Association. Elsevier Inc, 1–12. doi: 10.1016/j.jnma.2020.10.001. 33153755 PMC7607238

[ref5] Barrett CL (2020) Primary healthcare practitioners and patient blood management in Africa in the time of coronavirus disease 2019: safeguarding the blood supply. African Journal of Primary Health Care & Family Medicine 12, 3–5. doi: 10.4102/phcfm.v12i1.2457. PMC7284161 32501018

[ref6] Besigye IK, Mulowooza M and Namatovu J (2020) Coronavirus disease-2019 epidemic response in Uganda: the need to strengthen and engage primary healthcare. African Journal of Primary Health Care and Family Medicine 12, a2443. doi: 10.4102/PHCFM.V12I1.2443. PMC7284163 32501023

[ref7] Booth A, Omed RA and Naidoo M (2020) Analysis of a SARS-CoV-2 daily screening programme for healthcare workers at a district hospital in KwaZulu-Natal, a quality improvement initiative. African Journal of Primary Health Care and Family Medicine 12, 2–5. doi: 10.4102/PHCFM.V12I1.2525. PMC7479359 32896147

[ref8] Brey Z, Mash R, Goliath C and Roman D (2020) Home delivery of medication during Coronavirus disease 2019, Cape Town, South Africa: short report. African Journal of Primary Health Care and Family Medicine 12, a2449. doi: 10.4102/PHCFM.V12I1.2449. PMC7284162 32501022

[ref9] Chang B and Chiu T (2020) Ready for a long fight against the COVID-19 outbreak : an innovative model of tiered primary health care in Taiwan. BJGP Open 19–21. doi: 10.3399/bjgpopen20X101068. PMC7330205 32265184

[ref10] Corburn J, Vlahov D, Mberu B, Riley L, Caiaffa WT, Rashid SF, Ko A, Patel S, Jukur S, Martínez-Herrera E and Jayasinghe S (2020) Slum health: arresting COVID-19 and improving well-being in Urban informal settlements. Journal of Urban Health 97, 348–357. doi: 10.1007/s11524-020-00438-6. 32333243 PMC7182092

[ref11] David N and Mash R (2020) Community-based screening and testing for Coronavirus in Cape town, South Africa: short report. African Journal of Primary Health Care and Family Medicine 12, a2499. doi: 10.4102/PHCFM.V12I1.2499. PMC7284159 32501021

[ref12] Dyer O (2020) Covid-19 : Africa records over 10 000 cases as lockdowns take hold. BMJ 1439, 2020. doi: 10.1136/bmj.m1439. 32269023

[ref13] Furstenburg PP, Mukonkole SN, Kibamba CN, Kuiler A, Ngemntu N, Lahri SA, Van Hoving DJ, Moodley K and Erasmus E (2020) Emergency centre reorganization in preparation to the COVID-19 pandemic: a district hospital’s dynamic adaptation response. African Journal of Primary Health Care and Family Medicine 12, a2514. 10.4102/phcfm.v12i1.2514 PMC7564996 33054265

[ref14] Garg S, Basu S, Rustagi R and Borle A (2020) Primary health care facility preparedness for outpatient service provision during the COVID-19 pandemic in India: cross-sectional study. JMIR Public Health and Surveillance 6, e19927. doi: 10.2196/19927. 32452819 PMC7265797

[ref15] Goodyear-Smith F, Kinder K, Mannie C, Strydom S, Bazemore A and Phillips RL (2020) Relationship between the perceived strength of countries’ primary care system and COVID-19 mortality: an international survey study. BJGP Open 4, doi: 10.3399/bjgpopen20x101129. PMC7606144 32900707

[ref16] Haldane V, Zhang Z, Abbas RF, Dodd W, Lau LL, Kidd MR, Rouleau K, Zou G, Chao Z, Upshur RE and Walley J (2020) National primary care responses to COVID-19: a rapid review of the literature. BMJ Open 10, e041622. doi: 10.1136/bmjopen-2020-041622. PMC7725079 33293398

[ref17] Huston P, Campbell J, Russell G, Goodyear-Smith F, Phillips RL, van Weel C and Hogg W (2020) COVID-19 and primary care in six countries. BJGP Open 4, 1–6. doi: 10.3399/bjgpopen20X101128. PMC7606153 32900708

[ref18] Jenkins LS, Von Pressentin KB, Naidoo K and Schaefer R (2020) The evolving role of family physicians during the coronavirus disease 2019 crisis: an appreciative reflection. African Journal of Primary Health Care and Family Medicine 12, a2478. doi: 10.4102/PHCFM.V12I1.2478. PMC7343922 32634002

[ref19] Johnson O and Goronga T (2020) Why communities must be at the centre of the Coronavirus disease 2019 response: lessons from Ebola and human immunodeficiency virus in Africa. African Journal of Primary Health Care and Family Medicine 12, a2496. 10.4102/phcfm.v12i1.2496 PMC7343917 32634005

[ref20] Kusotera T and Nhengu TG (2020) Coronavirus-19 and malaria: the great mimics. African Journal of Primary Health Care and Family Medicine 12, 2–4. doi: 10.4102/PHCFM.V12I1.2501. PMC7479414 32787398

[ref21] Lim W and Wong W (2020) COVID-19 : notes From the Front Line, Singapore’s Primary Health Care Perspective. Annals of Family Medicine 18, 259–261. 32393562 10.1370/afm.2539 PMC7214001

[ref22] Loewenson R, Colvin CJ, Szabzon F, Das S, Khanna R, Coelho VS, Gansane Z, Yao S, Asibu WD, Rome N and Nolan E (2021) Beyond command and control: a rapid review of meaningful community-engaged responses to COVID-19. Global Public Health. Taylor & Francis, 0, 1–15. doi: 10.1080/17441692.2021.1900316. 33734007

[ref23] Madzimbamuto FD (2020) Ventilators are not the answer in Africa. African Journal of Primary Health Care and Family Medicine 12, 1–3. doi: 10.4102/PHCFM.V12I1.2517. PMC7433242 32787397

[ref24] Mash R, Goliath C and Perez G (2020) Re-organising primary health care to respond to the Coronavirus epidemic in Cape Town, South Africa. African Journal of Primary Health Care & Family Medicine 12, a2607. 10.4102/phcfm.v12i1.2607 PMC7669993 33181873

[ref25] Moolla MS, Broadhurst A, Parker MA, Parker A and Mowlana A (2020) Implementing a video call visit system in a coronavirus disease 2019 unit. African Journal of Primary Health Care and Family Medicine 12, a2637. 10.4102/phcfm.v12i1.2637 PMC7564671 33054264

[ref26] Motlhatlhedi K, Maotwe K, Bogatsu Y and Tsima B (2020) Coronavirus disease 2019 in Botswana: contributions from family physicians. African Journal of Primary Health Care and Family Medicine 12, 1–3. doi: 10.4102/PHCFM.V12I1.2497. PMC7433220 32787394

[ref27] Mulenga LB, Hines JZ, Fwoloshi S, Chirwa L, Siwingwa M, Yingst S, Wolkon A, Barradas DT, Favaloro J, Zulu JE and Banda D (2021) Articles prevalence of SARS-CoV-2 in six districts in Zambia in July, 2020: a cross-sectional cluster sample survey. The Lancet Global Health 9, e773–e781. 33711262 10.1016/S2214-109X(21)00053-X PMC8382844

[ref28] Nyasulu J and Pandya H (2020) The effects of coronavirus disease 2019 pandemic on the South African health system: a call to maintain essential health services. African Journal of Primary Health Care and Family Medicine 12, 1–5. doi: 10.4102/PHCFM.V12I1.2480. PMC7433230 32787396

[ref29] Olagundoye O, Enema O and Adebowale A (2020) Recommendations for a national Coronavirus disease 2019 response guideline for the care of older persons in Nigeria during and post-pandemic: a family physician’s perspective. African Journal of Primary Health Care and Family Medicine 12, 1–3. doi: 10.4102/PHCFM.V12I1.2512. PMC7479416 32787399

[ref30] Onagbiye SO, Mchiza ZJ, Bassett SH, Travill A and Eijnde BO (2020) Novel coronavirus and regular physical activity involvement: opinion. African Journal of Primary Health Care and Family Medicine 12, a2453. 10.4102/phcfm.v12i1.2453 PMC7284152 32501017

[ref31] Oseni TIA, Agbede RO, Fatusin BB and Odewale MA (2020) The role of the family physician in the fight against Coronavirus disease 2019 in Nigeria. African Journal of Primary Health Care & Family Medicine 12, a2492. 10.4102/phcfm.v12i1.2492 PMC7343949 32634004

[ref32] Porter J, Mash R and Preiser W (2020) Turnaround times – the Achilles ’ heel of community screening and testing in Cape Town, South Africa : a short report Laboratory turnaround times. African Journal of Primary Health Care & Family Medicine 12, a2624. 10.4102/phcfm.v12i1.2624 PMC7564763 33054266

[ref33] Rasanathan K and Evans TG (2020) Primary health care, the Declaration of Astana and COVID-19. Bulletin World Health Organ 98, 801–808. 10.2471/BLT.20.252932 PMC7607474 33177777

[ref34] Ray SC (2020) Editorial : covid-19 special collection. African Journal of Primary Health Care and Family Medicine 12, a2466. doi: 10.4102/phcfm.v12i1.2466. PMC7284157 32370525

[ref35] Reid S, Ras T and von Pressentin KB (2020) The Cape Town International Convention Centre from the inside : the family physicians ’ view of the ‘Hospital of Hope’. African Journal of Primary Health Care and Family Medicine 12, a2667. 10.4102/phcfm.v12i1.2667 PMC7670000 33181872

[ref36] Reuter H, Jenkins LS, De Jong M, Reid S and Vonk M (2020) Prohibiting alcohol sales during the coronavirus disease 2019 pandemic has positive effects on health services in South Africa Impact on the George Hospital. African Journal of Primary Health Care and Family Medicine 12, a2528. 10.4102/phcfm.v12i1.2528 PMC7433289 32787395

[ref37] Roman NV, Mthembu TG and Roman N (2020) Spiritual care – “A deeper immunity” – a response to Covid-19 pandemic Spiritual care in the South African. African Journal of Primary Health Care and Family Medicine 12, a2456. 10.4102/phcfm.v12i1.2456 PMC7343955 32634003

[ref38] Sarti TD, Lazarini WS, Fontenelle LF and Almeida AP (2020) What is the role of primary health care in the COVID-19 pandemic? Epidemiologia e serviços de saúde 29, 1–4. doi: 10.5123/S1679-49742020000200024. 32348404

[ref39] Sehoole TJ (2020) COVID-19: pandemic burden in Sub-Saharan Africa and the right to health-The need for advocacy in the face of growing privatisation. African Journal of Primary Health Care and Family Medicine 12, a2476. doi: 10.4102/PHCFM.V12I1.2476. PMC7564931 33054263

[ref40] Solomon G, Allie A, Fakier R, Tadmor D, Ashtiker K, Le Roux C, Omar J and Namane MK (2020) Family medicine internship support during the COVID-19 pandemic in Cape Town, South Africa – A narrative report. African Journal of Primary Health Care & Family Medicine 12, a2661. doi: 10.4102/phcfm.v12i1.2661. PMC7669940 33181875

[ref41] Tsima BM, Masupe T and Setlhare V (2020) Service-learning in response to the coronavirus disease 2019 pandemic: Emerging lessons from the department of family medicine and public health at the university of Botswana. African Journal of Primary Health Care and Family Medicine 12, 1–3. doi: 10.4102/PHCFM.V12I1.2455. PMC7284160 32501020

[ref42] Vandyck-Sey P, Amoh G, Essuman A and Lawson H (2020) Incidental finding of COVID-19 infection amongst staff at a primary care facility in Ghana. African Journal of Primary Health Care and Family Medicine 12, a2669. doi: 10.4102/PHCFM.V12I1.2669. PMC7564848 33054267

[ref43] WHO (no date) *Health promotion/social mobilization*. Available at: who.int/healthpromotion/social-mobilization/en/ (Accessed: 12 April 2021).

[ref44] World Health Organization (2020) WHO urges countries to move quickly to save lives from malaria in sub-Saharan Africa. Available at: www.who.int/news-room/detail/23-%0A04-2020-who-urges-countries-to-move-quickly-to-save-lives-from-malaria-in-subsaharan-afric (Accessed: 15 April 2020).

